# Persistence of fan-shaped keratocytes is a matrix-rigidity-dependent mechanism that requires α_5_β_1_ integrin engagement

**DOI:** 10.1038/srep34141

**Published:** 2016-09-28

**Authors:** Maryam Riaz, Marie Versaevel, Danahe Mohammed, Karine Glinel, Sylvain Gabriele

**Affiliations:** 1 Mechanobiology & Soft Matter Group, Laboratoire Interfaces et Fluides Complexes, Centre d’Innovation et de Recherche en Matériaux Polymères (CIRMAP), Research Institute for Biosciences, Université de Mons, 20, Place du Parc, B-7000 Mons, Belgium; 2 Institute of Condensed Matter & Nanosciences, Bio & Soft Matter division, Universite Catholique de Louvain, Croix du Sud 1, box L7.04.02, B-1348 Louvain-la-Neuve, Belgium

## Abstract

Despite the importance of matrix rigidity on cell functions, many aspects of the mechanosensing process in highly migratory cells remain elusive. Here, we studied the migration of highly motile keratocytes on culture substrates with similar biochemical properties and rigidities spanning the range between soft tissues (~kPa) and stiff culture substrates (~GPa). We show that morphology, polarization and persistence of motile keratocytes are regulated by the matrix stiffness over seven orders of magnitude, without changing the cell spreading area. Increasing the matrix rigidity leads to more F-actin in the lamellipodia and to the formation of mature contractile actomyosin fibers that control the cell rear retraction. Keratocytes remain rounded and form nascent adhesions on compliant substrates, whereas large and uniformly distributed focal adhesions are formed on fan-shaped keratocytes migrating on rigid surfaces. By combining poly-L-lysine, fibronectin and vitronectin coatings with selective blocking of α_v_β_3_ or α_5_β_1_ integrins, we show that α_V_β_3_ integrins permit the spreading of keratocytes but are not sufficient for polarization and rigidity sensing that require the engagement of α_5_β_1_ integrins. Our study demonstrates a matrix rigidity-dependent regulation of the directional persistence in motile keratocytes and refines the role of α_v_β_3_ and α_5_β_1_ integrins in the molecular clutch model.

Cell migration is an essential process in embryonic development, wound repair, and immune responses resulting from multiple interactions among intracellular organelles^1^. The directional movement of motile cells, which is critical to many physiological^2^ and pathological^3^ situations, is a highly integrated process guided by gradients of environmental cues. These cues may be diffusible or substrate–bounded, as in chemotaxis^4^ and haptotaxis^5^ respectively, or physical, including topography,^6^ electric fields^7^ or extracellular-matrix (ECM) rigidity^8^. The directed migration of a cell along an ECM-rigidity gradient, known as “durotaxis”, was originally observed in fibroblastic cells migrating along a soft-to-stiff interface^9^. Although durotaxis is thought to be critical to development of the nervous system^10^, epithelial-to-mesenchymal transition^11^, or cancer metastasis^12^, the cellular machinery used by motile cells to sense matrix rigidity and to migrate towards stiffer zones remains poorly understood.

Motile cells are thought to transmit forces to their surroundings by coupling their actin cytoskeleton to the ECM through adhesion sites^13^. Low motile cells, in general, exhibit highly organized actin stress fibers and larger spreading areas on stiff substrate, both leading to an accumulation of internal prestress^14^,^15^. Recent attentions to dynamic cellular processes on compliant environment have proposed the motor-clutch hypothesis^16^. In this model, cell adhesion molecules act as molecular clutches that create a frictional slippage interface modulating the degree of force transmission from actomyosin fibers. However, the role of the ECM rigidity in the regulation of motile cell properties such as cell shape, polarization, speed and trajectories is poorly characterized. In addition, the identification of the precise molecular interactions involved in the regulation of the molecular clutch on compliant matrices is critical to further understanding the rigidity-sensing mechanism of motile cells.

Among the receptor families responsible in regulating cell migration, integrins are the major trans-membrane receptors employed by cells to recognize, adhere and adapt to physico-chemical properties of their ECM. The 18 α and 8 β subunits assemble into 24 heterodimeric integrin complexes that exhibit varying affinity for ECM ligands and distinct signaling capabilities^17^. A particular attention has been placed on α_5_β_1_ and α_v_β_3_ receptors and their impact on cell migration^18^. Indeed, α_5_β_1_ and α_v_β_3_ integrin receptors bind respectively fibronectin (FN) and vitronectin (VN), both ECM glycoproteins containing the integrin-binding RGD sequence^19^. Interestingly, the expression profile of these integrins are often altered in pathological situations (e.g. angiogenesis, tumour metastasis or wound healing) and their individual role in cell migration remains controversy^20^ (Desgrosellier & Cheresh, 2010). Indeed, it has been suggested that β_1_ promotes random cell migration and β_3_ favors persistent migration^21^, while recently Missirlis *et al.* have suggested that directional migration on FN requires the engagement of both α_v_β_3_ and α_5_β_1_ integrins to the substrate^22^. As a consequence, determining the individual role of both integrin types in the mechanosensing mechanism of motile cells is a major open question in cell migration and may help to understand how the ECM rigidity regulates the morphology and the directional persistence of motile cells.

We addressed these issues in the context of epithelial keratocytes derived from fish skin, which represent an ideal model system for investigating the mechanisms of rigidity sensing in highly motile cells. Indeed, fish keratocytes are among the fastest moving animal cells and able to maintain nearly constant speed and direction during movement over long distances^23^. Motile keratocytes are characterized by a robust global shape determined by the mechanical feedback between the treadmilling actin network and the inextensible cell membrane^24^. Indeed, F-actin self-assembly at the plasma membrane pushes the membrane forward, whereas myosin motors, which are located at the rear cell edge pull F-actin rearward to generate F-actin retrograde flow. Large populations of keratocytes present a rich cell-to-cell variability that can be used as a key resource for mechanistic investigation regarding complex processes such as rigidity sensing^25^. To investigate how crawling cells sense and respond to matrix stiffness changes, we studied the migrating behaviour of individual keratocytes migrating on FN, poly-L-lysine (PLL) or VN-coated polymeric substrates that recapitulate a wide range of rigidities. We studied the morphology, polarization and directional persistence of motile keratocytes by varying the substrate stiffness over seven orders of magnitude. We then examined actin network organization, myosin localization and adhesion distribution for different matrix rigidities to elucidate the orchestration of cytoskeletal reorganization that leads to the adaptation of the cellular morphology to the change of rigidity. Finally, we sought to identify the molecular bonds involved in the rigidity-sensing mechanism of motile cells by focusing on α_5_β_1_ which is the major integrin receptor of FN that does not engage on VN and α_v_β_3_ integrin receptors that can bind both FN and VN.

## Results

### Matrix stiffness controls the morphology of highly motile cells

To investigate whether motility of crawling cells is affected by the stiffness of the substrate to which they adhere, we studied the behaviour of fish epithelial keratocytes on polymeric materials spanning a wide range of compliances to recapitulate the natural rigidity landscape in tissues^26^. We prepared polyacrylamide hydrogels (hydroxy-PAAm)^27^ of 1.5 kPa and 9 kPa, polydimethylsiloxane (PDMS)^28^ susbtrates of 110 kPa and 3 MPa, as well as glass substrates to mimic an infinite rigidity (~70 GPa). As introduced previously by some of us^29^,^30^ hydroxy-PAAm, PDMS and glass substrates were functionalized by microcontact printing with a similar amount of FN to avoid potential differences in ECM protein density. FN densities on all substrates were determined by immunofluorescence detection of labelled FN. We found a constant fluorescence intensity level across the different materials indicating uniform distribution of ligand density regardless the matrix stiffness (Fig. S1A in supplementary material). In addition, no statistical differences of FN density were found between the different matrices on the whole range of rigidities (Fig. S1B in supplementary material).

Recent works suggest that adherent cells may also adapt their response to the nature of the matrix^31^. Although the influence of the chemical nature of compliant matrices on cell fate is still under debate in the literature^32^, we investigated whether the nature of hydroxy-PAAm and PDMS matrices may lead to cell shape changes by plating fish keratocytes on hard hydroxy-PAAm hydrogels of 110 kPa and soft PDMS matrices of 9 kPa, both with a similar FN density. Our results indicated that differences in the chemical nature of hydroxy-PAAm and PDMS did not lead to significant differences in the shape of keratocytes (Fig. S1C in supplementary material), suggesting therefore that the modifications of the keratocyte morphology were only related to changes of the matrix Young’s modulus.

**Figure 1:**
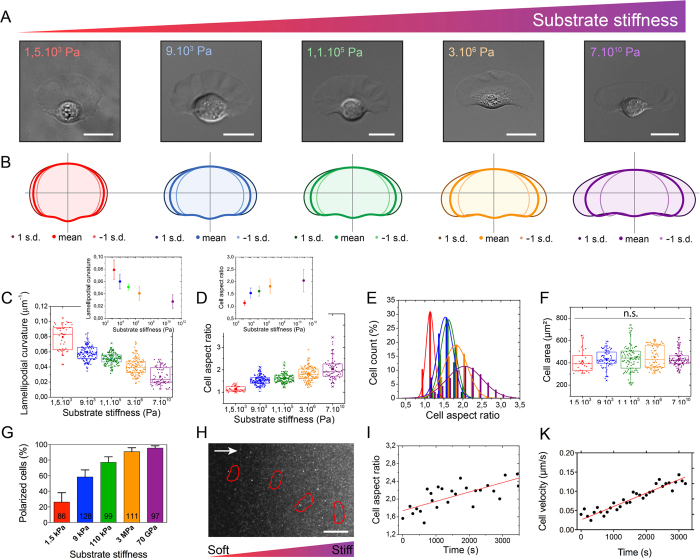
Matrix stiffness controls morphology and polarization of migrating cells. (**A**) Differential Interference Contrast (DIC) images of representative fish epithelial keratocytes migrating on culture substrates of different stiffnesses (E = 1.5 kPa in red, 9 kPa in blue, 110 kPa in green, 3 MPa in orange and 70 GPa in purple). (**B**) The variation of cell morphology, as determined by the analysis of aligned cell outlines, is shown for populations of crawling cells plated on substrates of various stiffnesses (80 ≤ n ≤ 160 for each population). The mean cell shape is presented in bold line for each population of cells with one standard deviation away from the mean in each direction. (**C**) Evolution of the lamellipodial curvature as a function of the substrate stiffness. The inset shows a semi-log scale. (**D**) Distribution of the aspect ratios of large populations of keratocytes plated on substrates of varying stiffness. The inset shows a semi-log scale. (**E**) Histogram of the cell count (%) as a function of the substrate stiffness. Lines are gaussian fits. (**F**) Distribution of the cell area of live keratocytes plated on different matrix rigidities (85 ≤ n ≤ 160 for each population). N.S indicates that no statistical difference was observed. (**G**) Histogram of the percentage of polarized cells within large populations of individual keratocytes plated on various matrix stiffnesses. The number of cells is indicated at the bottom of the bars. (**H**) The typical migration of a keratocyte from a soft (9 kPa) to a stiff (230 kPa) region is superimposed on the fluorescent image of beads embedded in the stiffer region of the hydrogel (see Movie S1). Red lines represent the cell boundary. White arrow indicates the direction of the gradient of rigidity from the softer (9 kPa) to the stiffer region (230 kPa). Scale bar is 50 μm. Temporal evolutions of (**I**) the cell aspect ratio and (**J**) the instantaneous cell velocity during the crossing event presented in (**H**).

Our results indicated that individual keratocytes assumed a variety of cell shapes according to the substrate rigidity (Fig. 1A), suggesting that motile keratocytes adapt their morphology to the matrix stiffness. As observed by SEM experiments, the major shape mode for FN-coated glass substrates was the highly polarized “canoe” shape as reported previously^33^, whereas the softer the matrix, the smaller the cell aspect ratio (Fig. S2A in supplementary material). To quantify the effect of matrix stiffness on keratocyte morphology, we first determined the principal modes of shape variations for large populations of cells plated 4 hours on different matrix stiffnesses. Principal component analysis of aligned outlines of keratocytes indicated that the standard deviation accounted for by each mode decreased on softer substrates (Fig. 1B), suggesting a lower shape variability on softer substrates. These shape variations were characterized by a two times decrease of the lamellipodial curvature (Fig. 1C).

To investigate further the effect of the matrix stiffness, we quantified the cell aspect ratio and the cell projected area for large populations of live keratocytes. Indeed, both parameters have been shown to be essential factors to capture morphological variations in fish keratocytes^34^. The cell aspect ratio increased non-monotonically with increasing matrix stiffness, exhibiting a mean aspect ratio of 1.13 ± 0.10 and 2.04 ± 0.46 on 1.5 kPa and 70 GPa substrates, respectively (Fig. 1D). Most of the individual keratocytes plated on soft substrates were rounded with a low variability in shape, whereas those plated on stiff matrices were elongated with a fan-shaped lamellipodium and a broader variability in shape (Fig. 1E). Taken together, these results indicate that the substrate stiffness mediates the cell morphology and can be considered as a valuable parameter to modulate the natural phenotypic variability in keratocyte populations^32^. Surprisingly, we found that variations of cell morphologies in response to the matrix stiffness did not imply a significant modification of the cellular area (Fig. 1F), which remained statistically similar over the wide range of stiffnesses. Further investigations demonstrated that the cell body area scaled linearly with the lamellipodial area (Fig. S2B in supplementary material), indicating that both cellular compartments mutually adapt their sizes independently of the matrix stiffness. Collectively, our findings suggest that the spreading responsiveness of low motile cells to matrix stiffness changes, as reported for different cell types^35^,^36^, cannot be generalized to epithelial motile cells such as keratocytes.

Then we quantified the fraction of polarized cells (i.e. motile cells) within large populations of keratocytes plated on different matrix stiffnesses. Our results demonstrate that the fraction of polarized cells increased significantly with increasing the matrix stiffness (Fig. 1G), suggesting that matrix stiffness changes affect the cell aspect ratio and the fraction of polarized cells.

Finally, we plated keratocytes on hydroxy-PAAm substrates with a gradient of rigidity ranging from 9 to 230 kPa (Fig. S1D and Movie S1) to observe the dynamic adaption of motile keratocytes in response to a gradient of rigidity. As observed previously for 3T3 fibroblasts, keratocytes migrated preferentially toward stiff substrates^9^ leading to an increase of the cell aspect ratio (Fig. 1I) and the cell velocity (Fig. 1K).

### Directional persistence is dependent on matrix stiffness

**Figure 2:**
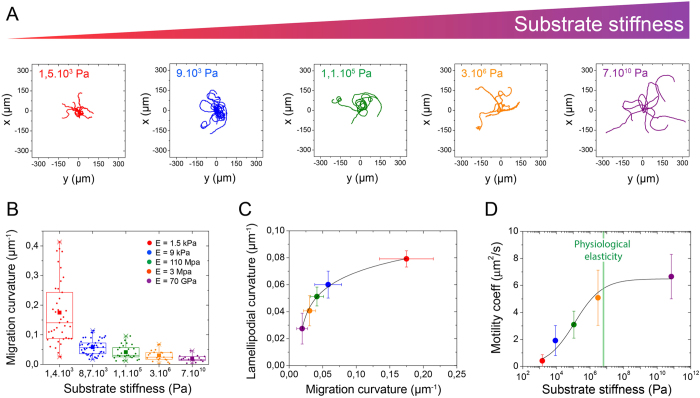
Migration is controlled by the matrix stiffness. (**A**) Superimposed migration trajectories of individual keratocytes plated for 30 min on flat substrates with various rigidities (red: 1.5 kPa, blue: 9 kPa, green: 110 kPa, orange: 3 MPa and purple: 70 GPa) and similar FN coatings. Initial positions of the crawling cells were superimposed at the origin for comparison. All plots range from −350 μm to + 350 μm on both the *x*- and *y*- axis. (**B**) Evolution of migration curvature as a function of the matrix stiffness. 85 ≤ *n* ≤ 160 for each population. (**C**) Evolution of the lamellipodial curvature as a function of the migration curvature. (**D**) Variation of the motility coefficient, *μ*, as a function of the substrate stiffness. The green mark represents the range of Young’s moduli of the internal side of fish scales^37^. Data are shown as mean ± s.d.

To gain more insight into the consequences of the morphological adaptation to stiffness, we next considered the effect of the matrix rigidity on the directional migration of crawling keratocytes. By using time-lapse microscopy in DIC mode, we tracked in *x,y* coordinates the cell body of crawling cells migrating on different matrix rigidities. As shown in Fig. 2A, the length of the typical trajectories of keratocytes was qualitatively longer with increasing matrix stiffness. We found that the migration curvature decreased abruptly from 0.17 ± 0.04 to 0.06 ± 0.02 for cells plated on 1.5 kPa and 9 kPa substrates, respectively, then slowly to reach a value of 0.02 ± 0.01 for cells migrating on 70 GPa substrates (Fig. 2B and Fig. S2C in supplementary material). These results show that rounded keratocytes on soft substrates exhibited more curved trajectories than polarized cells on stiff substrates. We then examined whether the curvature of migration was related to the cell morphology. We found that canoe-shaped keratocytes migrating on stiffer substrates exhibited lowest lamellipodial and migration curvatures, whereas softer substrates were characterized by highest values of lamellipodial and migration curvatures (Fig. 2C), suggesting that the morphological adaptation of crawling keratocytes to the matrix stiffness is tightly coupled to a modification of their migrating behaviour. To examine more quantitatively the effect of the matrix stiffness on cell motility, we calculated the mean square displacement (MSD) versus time for large number of cells (85 ≤ *n* ≤ 160) plated on different matrix rigidities. Using MSD curves, we determined the translocation speed, S, (Fig. S3A in supplementary material) and the persistent time, T (Fig. S3B in supplementary material). According to S and T mean values obtained for each rigidities, we determined a motility coefficient, μ, that represents the area explored by motile cells by time unit and defined as^37^: μ = 1/2S^2^T. As shown in Fig. 2D, the motility coefficient, μ, increased significantly with the matrix stiffness, suggesting that increasing matrix rigidity permits to crawling keratocytes to explore larger areas. Crawling cells on stiffer matrix were described by μ = 6.8 ± 1.5 μm^2^/s (E = 70 GPa), which was more than 15 times higher than the motility coefficient on the softer microenvironment (μ = 0.4 ± 0.2 μm^2^/s for E = 1.5 kPa). Interestingly, the motility coefficient reached a plateau for matrix rigidities around ~600 MPa (Fig. 2D), which correspond to the physiological range of stiffnesses of the keratocyte environment, as characterized by the Young’s modulus of the internal side of fish scales^38^.

### Matrix stiffness modulates actin and myosin distribution patterns

**Figure 3:**
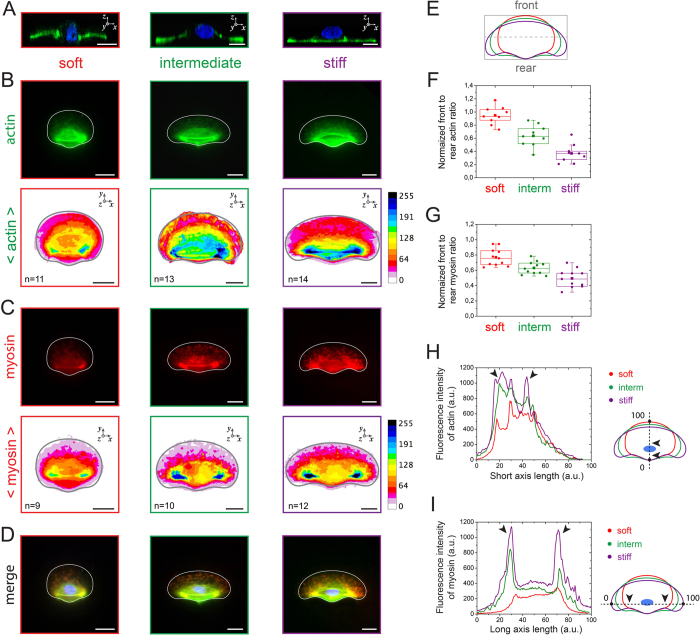
Matrix stiffness affects actin and myosin distribution patterns. Immunostained images of individual keratocytes plated on soft (left column, in red), intermediate (center column, in green), and stiff (right column, in purple) culture substrates. (**A**) Cross-sectional confocal views (*xz*) and (**B**) normal views (*xy*) of keratocytes labeled for actin with fluorescent phalloidin. The color-coded average distribution of actin is shown for n = 11 (soft), n = 13 (intermediate) and n = 14 cells (stiff). (**C**) Normal views of keratocytes immunolabeled for myosin and color-coded average distribution of myosin for n = 9 (soft), n = 10 (intermediate) and n = 12 cells (stiff). (**D**) Merge images of (**B**) and (**C**). The front and rear division schematically presented in (**E**) was used to estimate the front to rear actin ratio (**F**) and myosin ration (**G**) for soft (in red), intermediate (in green) and stiff (in purple) substrates. (**H**) The distribution of actin was measured along a line from the cell rear (point 0) to the cell front (point 100) for keratocytes plated on soft (in red), intermediate (in green) and stiff (in purple) culture substrates. (**I**) The distribution of myosin was measured from the left side (point 0) to the right side (point 100) for crawling cells plated on soft (red), intermediate (green) and stiff (purple) culture substrates. Black arrows in (**H**) and (**J**) indicate the highest fluorescent signals. Scale bars are 5 μm.

To explore the underlying cytoskeletal mechanism of rigidity-induced polarization of keratocytes, we characterized by confocal microscopy the spatial distribution of actin (Fig. 3A,B) and myosin II (Fig. 3C) in keratocytes plated on soft (E = 1.5 kPa, Movie S2 in supplementary material), intermediate (E = 110 kPa, Movie S3 in supplementary material) and stiff matrices (E = 70 GPa, Movie S4 in supplementary material). Cross-sectional views (Fig. 3A) indicated that the lamellipodia of keratocytes migrating on soft and intermediate matrices was curved on the *x,z* plane (Movie S2 in supplementary material), whereas it remained perfectly flat on rigid substrates, demonstrating that cells were able to deform soft underlying substrates. Immunostained images of F-actin indicated high fluorescent signals at the cell rear with increasing substrate rigidity, suggesting the formation of actin stress fibers normally to the direction of migration (Fig. 3B and Movie S4 in supplementary material). Immunostained images of myosin II showed that increasing the substrate rigidity leads to high fluorescent signals localized at the cell rear on both sides of the cell body (Fig. 3C). Interestingly, merge images of actin and myosin II indicated that both signals colocalize at the cell rear (Fig. 3D).

We next confirmed these observations by quantifying the front to rear actin and myosin ratios by dividing keratocytes in two parts (Fig. 3E). Our results confirmed a significant accumulation of actin (Fig. 3F) and myosin (Fig. 3G) in the rear part of the motile cells, whereas it is interesting to note that keratocytes on soft substrates exhibit an homogeneous spatial distribution of actin and myosin. Plot profiles of immunostained images showed that the width of branched F-actin in the lamellipodia became significantly larger with increasing the matrix stiffness (Fig. 3H), whereas myosin II accumulated significantly at the rear part of the cell, leading to large and intense areas of myosin II located at both sides of the cell body on stiff substrates (Fig. 3I, black arrows)^39^.

Our results demonstrated that increasing the matrix rigidity leads to more F-actin in the lamellipodia, which corresponds to the main driving force for keratocyte locomotion^40^, and to the formation of mature contractile actomyosin fibers that control the cell rear retraction by generating inward contractile forces^41^.

Assuming that the molecular clutch model predicts two distinct regimes: (i) an oscillatory “load-and-fail” dynamics associated with high traction forces on soft substrates and (ii) a “frictional slippage” associated with low traction forces on stiff substrates, we next investigated whether the matrix stiffness modulates the adhesion strength in order to explain why keratocytes fail to elongate and polarize on soft substrates.

### Mature focal adhesions are promoted on rigid substrates

**Figure 4:**
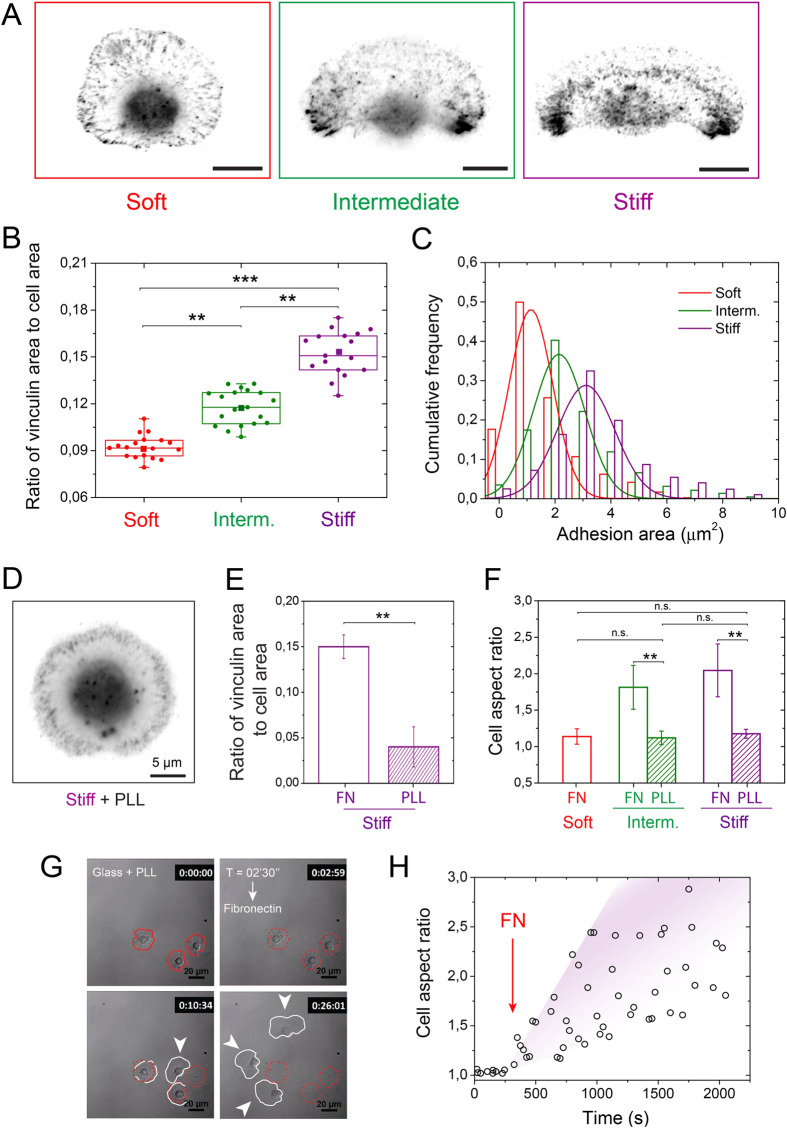
Cell-substrate adhesions are modulated by matrix stiffness. (**A**) Typical inverted images of keratocytes plated on soft (left, in red), intermediate (center, in green), and stiff (right, in purple) culture substrates and immunolabeled for vinculin. (**B**) Evolution of the ratio of vinculin area to cell area with the substrate rigidity. (**C**) Cumulative frequencies of the adhesion areas for soft (in red), intermediate (in green) and stiff (in purple) matrices. Solid lines are gaussian fits. (**D**) Inverted image of a keratocyte plated on a stiff substrate coated with PLL and immunolabeled for vinculin. (**E**) Ratio of the vinculin area to the cell area measured for keratocytes plated on stiff substrates coated with PLL (n = 16) and FN (n = 19). (**F**) Evolution of the cell aspect ratio on soft (in red), intermediate (in green) and stiff (in purple) substrates coated with FN (plain bars) and PLL (hashed bars). (**G**) Image sequence of crawling cells plated on a stiff substrate coated with PLL that polarized and migrated after the addition of fibronectin (t = 02 min 30 sec.) in the culture media. (**H**) Temporal evolution of the aspect ratio of crawling cells plated on a stiff substrate coated with PLL in response to the addition of fibronectin (red arrow) in the culture media. *p < 0.05, **p < 0.01, ***p < 0.001 and n.s. not significant.

We next immunostained keratocytes plated 4 hours on soft (E = 1.5 kPa), intermediate (E = 110 kPa) and stiff (E = 70 GPa) matrices for vinculin, which is one of the linker proteins between actin filaments and transmembrane integrins. Qualitatively, vinculin-containing adhesions were homogeneously distributed around the cell periphery in rounded cells migrating on soft substrates, whereas vinculin gradually concentrated at the two extremities of the rear part of polarized cells with increasing the substrate rigidity (Fig. 4A). We thresholded immunostained images and applied a watershed segmentation algorithm to quantify the size distribution of vinculin-containing focal adhesion by separating neighbouring structures according to the intensity valley between them. After quantification, we found that the ratio of vinculin area to cell area increased from 0.092 ± 0.017 on soft substrates to 0.117 ± 0.015 and 0.153 ± 0.022 on intermediate and stiff substrates, respectively (Fig. 4B), suggesting a stronger cell-matrix adhesion on rigid substrates. Additionally, the size distribution of vinculin sites demonstrated that the percentage of small nascent adhesions (~1 μm^2^) was significantly more elevated on soft matrices, whereas mature focal adhesions (3–4 μm^2^) dominated on stiff substrates (Fig. 4C).

In light of our previous results on actomyosin, this observation suggests that adhesion strength is too weak on soft substrates to resist to contractile forces, leading to round-shaped cells. In contrary, increasing the matrix rigidity promotes the formation of mature focal adhesions that permit keratocytes to contract their rear side, leading to a larger fraction of polarized cells (Fig. 1G).

We next investigated the role of integrin-mediated focal adhesions on the keratocyte morphology by coating intermediate and stiff substrates with poly-L-lysine (PLL) that does not allow specific integrin engagements^42^. Despite the large rigidity of glass substrates, keratocytes remained unpolarised on PLL-coated glass substrates (94% of stationary cells, Fig. 4D) and exhibited a very low amount of vinculin (Fig. 4E). Statistical tests indicated that keratocytes plated on intermediate and stiff PLL-coated substrates were rounded with a low mean cell aspect ratio (1.12 ± 0.09 for intermediate and 1.09 ± 0.06 for stiff), as observed for keratocytes on FN-coated soft matrices (Fig. 4F), suggesting an integrin-dependent rigidity sensing mechanism for cell polarization. To confirm this hypothesis, we carried out motility experiments on stiff PLL-coated substrates with an addition of FN into the culture media. At the beginning of the experiment, cells were rounded and remained stationary (Fig. 4G). After adding FN in the culture media at *t* = 150 sec., keratocytes started to move from 510–1120 sec. (Fig. 4g and Movie S5 in supplementary material). Interestingly, the time range 500–1000 sec. required to observe cell displacements corresponded to the time needed for diffusion and adsorption of FN on PLL-coated glass substrates (Fig. S3C). Cell migration initiated by FN adsorption was accompanied by a significant increase of the cell aspect ratio (Fig. 4H), demonstrating that specific cell-substrate interactions mediated by integrins are required for the rigidity sensing mechanism of keratocytes.

Collectively, these data demonstrate that matrix stiffness mediates the formation of focal adhesions in migrating keratocytes and underline the importance to determine the type of the integrin receptors that mediate rigidity sensing.

### The rigidity sensing mechanism in highly motile cells requires the engagement of α_5_β_1_ integrins

We next sought to determine the type of integrin receptors involved in the rigidity sensing mechanism by focusing on two receptors of the ECM protein FN: α_5_β_1_ and α_V_β_3_ integrins.

**Figure 5:**
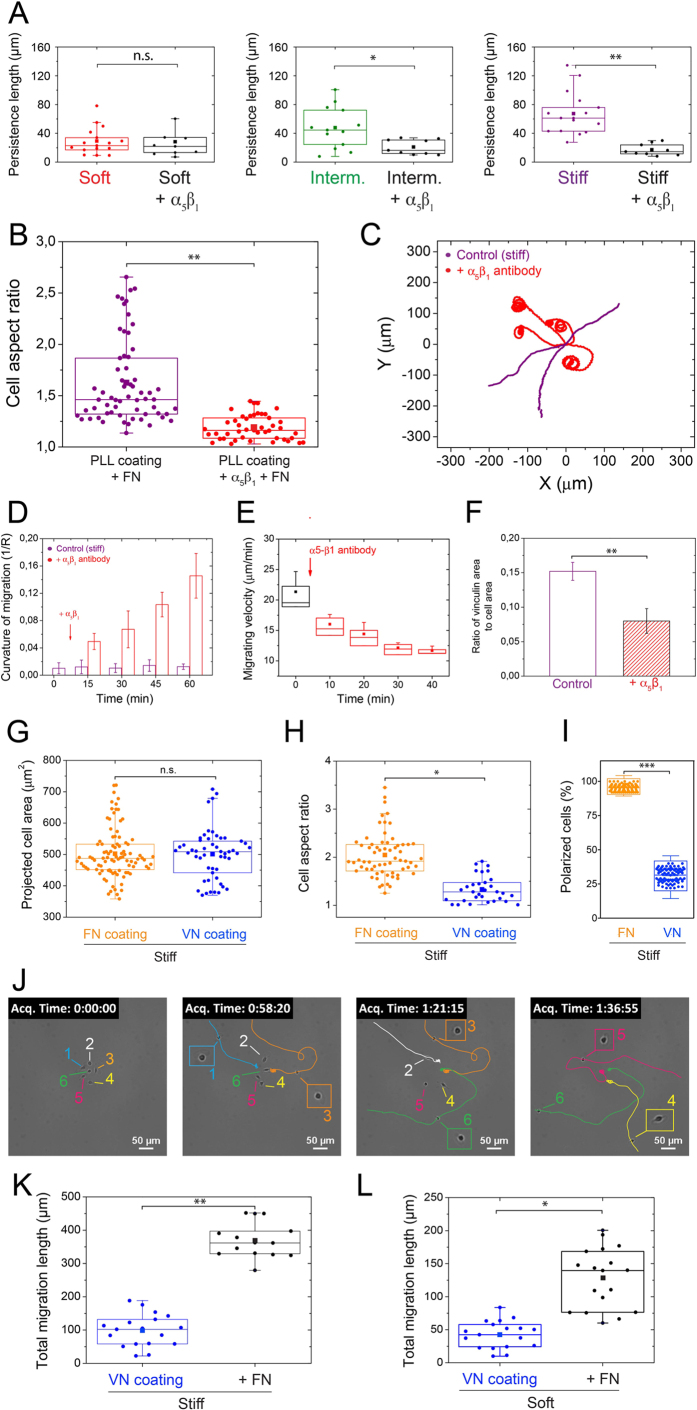
α5β1 integrin engagement is required for keratocyte mechanosensing. (**A**) Effect of a α_5_β_1_ antibody treatment on the persistence length of individual keratocytes plated on soft (in red), intermediate (in green) and stiff (in purple) FN-coated substrates. (**B**) Comparison of the cell aspect ratio of keratocytes plated on PLL-coated stiff substrates after addition of FN in the culture media (in purple) and with the addition of FN in the culture media of keratocytes treated with α_5_β_1_ antibody and plated on PLL-coated stiff substrates (in purple). (**C**) Typical trajectories described by keratocytes on stiff substrate after a α_5_β_1_ antibody treatment (in red). Temporal variation of (**D**) the migration curvature (**E**) the migrating velocity of keratocytes plated on FN-coated stiff substrates and treated with a α_5_β_1_ antibody. (**F**) Ratio of the vinculin area to the cell area measured for keratocytes migrating on stiff FN-coated substrates (in purple) and treated with α_5_β_1_ antibody (dashed red bar). (**G**) Projected area, (**H**) cell aspect ratio and (**I**) polarized fraction of individual keratocytes plated on stiff substrates coated with FN (in orange) and VN (in blue). (**J**) Sequence of the migration of 6 keratocytes plated on a stiff VN-coated surface in response to FN added in the culture media at t = 42 min 50 sec. Evolution of the total migration length of keratocytes migrating on (**K**) soft and (**L**) stiff substrates coated with VN after addition of FN in the culture media. *p < 0.05, **p < 0.01 and n.s. not significant.

To this end, we first quantified the persistence length on soft, intermediate and stiff substrates of keratocytes treated with an antibody against α_5_β_1_. As shown in Fig. 5A, α_5_β_1_ antibody treatment decreased drastically the persistence length, regardless the matrix stiffness, underlying the importance of α_5_β_1_ integrin in rigidity-sensing. To confirm this result, we quantified the cell aspect ratio of α_5_β_1_-treated cells plated on stiff PLL-coated substrates in response to an addition of FN in the culture media. Our results showed that α_5_β_1_-treated keratocytes adopted a rounded shape (mean aspect ratio of 1.18 ± 0.10) and remained static on PLL-coated stiff substrates, even in the presence of FN added in the culture media (Fig. 5B). Next, we observed the motile behaviour of keratocytes plated on FN-coated stiff substrates in response to the addition of α_5_β_1_ antibody in the culture media. Initially, polarized cells migrated at ~21 ± 4 μm/min and described a persistent trajectory (Fig. 5C, in purple). After addition of α_5_β_1_ antibody in the culture media, the curvature of migration increased significantly with time to reach 0.15 ± 0.03 μm^−1^ at ~55 min. of treatment (Fig. 5C,D and Movie S6), whereas the migrating velocity decreased to 11.61 ± 0.82 μm/min (Fig. 5E). Furthermore, quantification of vinculin indicated that the amount of focal adhesions was statistically two times lower in α_5_β_1_-antibody-treated cells (Fig. 5F). Taken together, these results demonstrate that the engagement of α_5_β_1_ integrin is necessary for the rigidity-sensing mechanism of keratocytes.

However, the mechanistic role of α_v_β_3_ integrins in the rigidity-sensing mechanism is as yet undefined. To address this issue, we presented keratocytes with substrates coated with VN. Indeed α_5_β_1_ integrin receptors do not recognize VN, whereas α_V_β_3_ can bind both FN and VN. Our strategy permits therefore to study how different ECM receptor engagement affects the migration of keratocytes avoiding genetic manipulation. Keratocytes plated on VN-coated stiff substrates exhibited statistically similar spreading areas than those plated on FN (Fig. 5G) but a lower aspect ratio (1.29 ± 0.18, Fig. 5H), suggesting that α_v_β_3_ integrins were required for cell spreading but not sufficient for cell shape adaptation to the matrix stiffness. In addition, we found that the fraction of polarized cells on stiff VN-coated substrates was very low (Fig. 5I) and the tracking of keratocytes on VN-coated stiff substrates indicated that cells remained stationary for very long periods of time. We then added FN into the culture media (t = 43 min 05 sec.) of keratocytes plated on stiff VN-coated substrates and we observed that keratocytes initially stationary started to migrate (Fig. 5J and Movie S7 in supplementary material). Furthermore, we confirmed this observation by quantifying the total migration length of keratocytes on soft (Fig. 5K) and stiff (Fig. 5L) VN-coated substrates, which was significantly higher by adding FN in the culture media.

Taken together, our results demonstrate that α_V_β_3_ integrins permit the spreading of keratocytes but are not sufficient for polarization and rigidity sensing that require the engagement of α_5_β_1_ integrins.

## Discussion

The shape of motile cells is determined by many dynamical processes that emerge from the interaction of different cell components such as the cytoskeleton, the cell membrane and the cell-substrate adhesions. Previous reports have demonstrated a biphasic dependence of fibroblast and keratocyte migration rates on ECM ligand density^33^,^43^. Low protein density fails to generate mature cell-substrate adhesions, whereas high protein density inhibits cell tail retraction, leading to slow migration rate. As a consequence, optimal migration rate on two-dimensional substrates occurs at intermediate levels of cell-ligand density. By maintaining constant the cell-ligand density in the optimal range and varying independently the substrate stiffness over seven orders of magnitude, our results demonstrate that the migrating phenotype of crawling keratocytes is mediated by the matrix stiffness in a continuous and progressive way. Indeed, our findings show that increasing matrix stiffness leads to larger fraction of polarized keratocytes within a population that adopt a persistent migration. As a consequence, our results reveal that changing matrix stiffness alone can modulate the natural phenotypic variability of large populations of keratocytes. Interestingly, we found that the most efficient migrating behaviour is obtained for matrix stiffness from ~600 MPa, which corresponds to the stiffness range of the natural environment of fish epithelial keratocytes^37^.

Our results show that the rigidity-dependent cell shapes are largely determined by modifications of the spatial distribution of actin and myosin spatial, which have been already identified as responsible of the spontaneous symmetry breaking in keratocytes^44^. Indeed, increasing the matrix rigidity leads to the formation of thick actin fibers at the rear cell that are oriented normally to the direction of migration and colocalize with large accumulation of myosin on stiff substrates. Matrix stiffness modulates the polarization of keratocytes through the formation of contractile actomyosin fibers that exert inward forces at the rear cell.

Previous reports have shown that contractile stresses generated by the actomyosin system are transmitted to the substrates through adhesion sites, providing the necessary forces required for cell propulsion^13^,^45^. Emerging evidence suggests that adhesion sites are formed near the front of the cell, then grow into mature focal adhesions found at the rear part and finally disassemble as the cell advances^46^. Our results show that keratocytes on soft substrates form small nascent adhesions homogeneously distributed along the cell periphery, whereas stiff substrates promote the formation of mature focal adhesions at the rear, which are located on both sides of the cell body. Because adhesion maturation is driven by myosin II contractility^47^, it is predictable that stiffness-dependent distribution of myosin II could affect the formation of mature focal adhesions, which in turn regulates cell motility. In addition, it has been demonstrated that actomyosin tension is required for the growth of FAs at the leading edge^48^ and for the retraction of the cell rear through FA disassembly^49^. Our findings confirm these results and highlight the role of the matrix stiffness in mediating cell-substrate interactions through the regulation of the actomyosin activity.

The force transmission between the actin flow and cell adhesion complexes is often viewed as a molecular clutch that is either engaged or disengaged^50^. When an actin filament is fixed with respect to the substrate (i.e., a clutch is engaged), there is no slippage between the actin cytoskeleton and the substrate, leading to a productive cell movement. In contrast, when the clutch is disengaged, the slippage that occurs between the actin network and the adhesion complexes increases the retrograde flow and decreases the protusion rate. Recently, study of neuronal cells^51^ refined the molecular clutch model by taking the effect of the matrix stiffness into account. Chan and coworkers introduced two different modes of the adhesive machinery that consider the switching between load and fail dynamics and frictional slippage in response to the matrix rigidity. Despite these recent efforts, the role of different classes of αβ-integrins binding to FN in the rigidity sensing mechanism of motile cells is still elusive.

**Figure 6:**
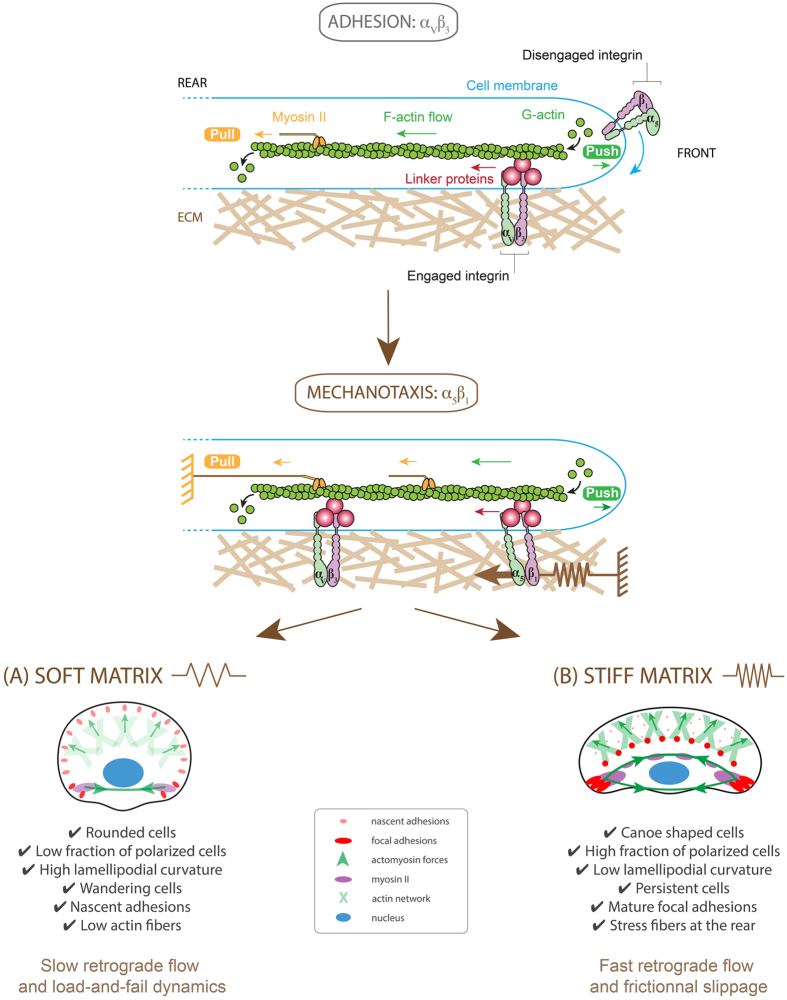
Schematic of the role of α_5_β_1_ and α_V_β_3_ integrins in the focal adhesion clutch model. Integrins are coupled to F-actin via linker proteins, such as vinculin, talin and paxilin, that move backward (red arrows) in response to pushing forces (in green) exerted by actin polymerization and pulling forces (in orange) exerted by actomyosin contractility. Cell spreading requires only α_V_β_3_ integrin engagement. (**A**) On soft matrices, keratocytes have a low aspect ratio, are characterized by a low fraction of polarized cells, form nascent adhesions located at the cell periphery, have a low persistence and a high lamellipodial curvature. (**B**) On stiff matrices, keratocytes have a high aspect ratio with mature focal adhesions, have a high fraction of polarized cells, a high persistence and a low lamellipodial curvature.

We addressed this question by studying the role of α_5_β_1_ and α_V_β_3_ integrins which are involved in the formation of linkages with adhesion proteins and F-actin^52^. Recently, Roca-Cusachs and coworkers reported that α_5_β_1_ and α_V_β_3_ integrins might have opposite mechanical roles^53^. Indeed, while high matrix forces are primarily supported by clustered α_5_β_1_ integrins that provide strong molecular bonds to resist high forces, less stable links to α_V_β_3_ integrins initiate mechanotransduction. In addition, previous reports have shown that α_5_β_1_ integrins colocalize initially with α_V_β_3_ integrins in focal contacts at the cell edge but subsequently translocate toward cell interior, which corresponds to less dynamic zones. Our results are consistent with this observation and help interpret previous findings in the context of mechanosensing (Fig. 6). Indeed, our data indicate that keratocytes spread on VN-coated substrates by using α_V_β_3_ integrins but failed to migrate, regardless the matrix stiffness. We have demonstrated that addition of FN in the culture medium induced cell polarization and migration by allowing keratocytes to engage α_5_β_1_ integrins. The key role of α_5_β_1_ in the rigidity sensing mechanism was confirmed by quantifying the persistence length of α_5_β_1_ antibody treated keratocytes plated on FN-coated substrates of varying rigidities. Our conclusions are in line with recent studies reporting β_1_ integrin retrograde transport as essential to maintain persistent cell migration^54^ and previous work demonstrated that cells exhibit low directional persistence on patterned surfaces of VN. In addition, our mechanistic model presented in Fig. 6 is also consistent with binding/unbinding rates of α_5_β_1_ and α_V_β_3_ integrins, as reported recently^55^. Indeed, the fast rate of binding/unbinding of α_V_β_3_ links is particularly appropriated for spreading events, whereas the slower dynamics of α_5_β_1_ enable adhesion reinforcement.

## Materials and Methods

### Keratocyte culture and reagents

Keratocytes were cultured from the scales of Central American cichlid Hypsophrys Nicaraguensis. Keratocytes were sandwiched between two 25 mm diameter glass coverslips and cultured in Leibovitz’s Media (L-15) supplemented with 10% FBS, 1% antibiotic-antimycotic, 14.2 mM HEPES and 30% deionized water at room temperature for 12 hours. Keratocytes were then detached from the glass coverslip by incubating with a trypsin solution (1 ml per glass slide) for 5 minutes and resuspended in 4 ml of L-15 Leibovitz complete medium. Suspended cells were then transferred to FN-coated substrates. All experiments were made between 2 and 8 hours after cell seeding.

### Preparation of polycarylamide hydrogels

Acrylamide (AAm), bisacrylamide (bis-AAm) and N-hydroxyethylacrylamide monomers (HEA) containing a primary hydroxyl group were copolymerized to form a hydrophilic network of polyacrylamide with hydroxyl groups by random radical polymerization, as previously described^26^. Briefly, hydroxy-PAAm hydrogels were synthesized on circular glass coverslips of 25 mm in diameter that were cleaned with 0.1 M NaOH (Sigma, Saint-Louis, MO) solution during 5 min and then rinsed abundantly (20 min under agitation) with deionized water. Cleaned coverslips were treated during one hour with 3-(trimethoxysilyl)propyl acrylate (Sigma, Saint-Louis, MO) to promote strong adhesion between the hydroxy-PAAm gel and glass coverslips and then dried under a nitrogen flow. In a 15 mL Eppendorf tube, 400 μL acrylamide, 40% w/w in HEPES (AAm, Sigma, Belgium), 50 or 250 μL N,N’-methylenebisacrylamide, 2% w/w in HEPES (BisAAm, Sigma, Saint-Louis, MO), and 1065 μL N-hydroxyethylacrylamide monomers (65 mg.mL^−1^ in HEPES, Sigma, Saint-Louis, MO) were mixed and the desired volume of a solution of 4-(2-hydroxyethyl)-1-piperazineethanesulfonic acid 50 mM (HEPES, Sigma, Saint-Louis, MO) was added to reach a final volume of 5 mL and a young modulus of 1.5 kPa or 9 kPa, respectively. Very stiff hydrogels were prepared by using a 29:1 ratio of monomer to crosslinker, as previously described^56^. We prepared a mixture with 685 mg or 910 mg acrylamide, 65 mg of N-hydroxyethylacrylamide, 25.9 or 34.5 mg of N,N’-methylenebisacrylamide and a HEPES solution to reach a final volume of 5 ml and final Young’s moduli of 110 kPa or 230 kPa. After degassing the mixture during 20 min under vacuum, the polymerization was started by adding 2.5 μL of N,N,N’,N’-tetramethylethylenediamine (TEMED, Sigma, Saint-Louis, MO) and 25 μL of ammonium persulfate solution (APS, 100 mg/ml w/w in deionized water, Sigma, Saint-Louis, MO). A volume of 50 μL of this mixture was deposited on a 25 mm diameter glass coverslip and then a 22 mm diameter glass coverslip was placed upon the solution. After 30 min, the polymerization was completed and the 22 mm diameter coverslip was gently removed to obtain ~150 μm thick hydroxy-PAAm hydrogels. Finally, hydroxy-PAAm hydrogels were washed three times in sterile deionized water and stored at 4 °C in sterile phosphate buffer saline (PBS).

### Preparation of polyacrylamide hydrogel with gradient in stiffness

Hydroxy-PAAm hydrogels with stiffness gradients between 9 kPa and 230 kPa were prepared by using a method introduced by Lo and coworkers^9^. Stiffness gradients were made by juxtaposing two drops of 20 μl of different acrylamide and bisacrylamide concentrations to obtain 9 and 230 kPa on a 25-mm activated circular coverslip. The unpolymerized solution of the stiffest gel contained 0.04 μl of fluorescent beads of 0.2 μm in diameter (Fluospheres, Invitrogen). This ensured that any possible stiffening effects of the beads would be on the stiff side of the gel. The two drops were mixed by applying gently a 25-mm circular coverslip. Gradient gels were allowed to polymerize for 30 min, then the coverslip was removed and the resulting hydrogel with a stiffness gradient was washed twice with PBS.

### Preparation of polydimethylsiloxane elastomers

Polydimethylsiloxane (PDMS) substrates of 9 kPa, 110 KPa and 3 MPa were prepared from the commercially available Sylgard 184 silicone elastomer kit (Dow Corning, Midland, MI) by mixing the base and the curing agent in varying ratios, as described previously^27^. Specifically, PDMS with base to crosslinker (w/w) ratio of 52:1, 35:1 and 10:1 were prepared to obtain 9 kPa, 110 kPa and 3 MPa Young’s modulus, respectively. Pre-polymer solutions were mixed thoroughly for at least 5 minutes, degassed, and spin-coated at 5000 rpm on 25 mm glass coverslips. PDMS was then cured for 2–3 hours at 60 °C. Samples were stored at room temperature in a vacuum desiccator.

### Measurement of polyacrylamide and polydimethylsiloxane stiffness

The stiffness (Young’s modulus) of hydroxy-PAAm hydrogels and PDMS elastomers was measured by DMA (Dynamic Mechanical Analysis, Mettler Toledo DMA/SDTA 861e, Switzerland). Briefly, DMA analysis in compression mode was undertaken on circular cylindrical samples of 15 mm in diameter and height of 10 mm. Samples were sandwiched between two parallel plates and an oscillating strain of maximum amplitude of 10% was applied. The stress needed to deform the cylindrical samples (n = 13) was measured over a frequency range of 0.1–10 Hz. During compression testing, a settling time of approximately one minute was used to achieve a stable measurement of the storage modulus at each frequency.

### PDMS microstamps

Flat PDMS microstamps were prepared by casting a 10:1 (w/w) degassed mixture of PDMS prepolymer and curing agent (Sylgard 184, Dow Corning) on a silicon wafer, which was passivated with fluorosilane (tridecafluoro-1,1,2,2-tetrahydrooctyl-1-trichlorosilane, Gelest) in a vacuum to facilitate the removal of the PDMS layer. After curing overnight at 65 °C, flat PDMS microstamps were peeled off from the silicon master, washed with ethanol and made hydrophilic by exposure to ultraviolet ozone (UV/O_3_). Activated PDMS microstamps were finally coated with a sterile solution of FN for 1 hour at room temperature.

### Microcontact printing

PA, PDMS and activated glass coverslips (170 μm thick and ~70 GPa) substrates were homogeneously functionalized by microprinting using a flat PDMS substrate incubated one hour with a 50 μg/ml solution of FN or VN from human plasma. PLL coatings were obtained by incubating glass substrates with a 0.01% solution of poly-L-lysine (Sigma-Aldrich) overnight at 4 °C.

### Immunocytochemistry

Intracellular components were made visible using fluorescent staining techniques. Briefly, fish keratocytes were rinsed 3 times with PBS (pH ~7.4), fixed and permeabilized with a first incubation of 1 min in 0.035% glutaraldehyde and 0.1% triton X-100 followed by a rinse in PBS and a second 10 min incubation in a 0.14% solution of glutaraldehyde. Coverslips were rinsed extensively with PBS and then incubated for 45 min at 37 °C with Alexa Fluor 488 phalloidin (Invitrogen, 1:200) for staining filamentous actin, DAPI^57^ (Invitrogen, 1:200) to visualize the nuclei, and a primary antibody (anti-vinculin antibody produced in mouse, Sigma-Aldrich, HVIN-1 clone, 1:200) or anti-myosin light chain antibody produced in rabbit (Abcam, ab2480, 1:300). A tetramethylrhodamine-labelled secondary antibody (goat anti-rabbit 1:400 or goat anti-mouse 1:200, Sigma-Aldrich) was then used for 45 min at 37 °C. Slides were mounted in Slow Fade Gold Antifade (Molecular Probes, Invitrogen).

### Pharmacological treatments

An antibody anti-integrin α_5_β_1_ was used to prevent cell adhesion to fibronectin (Antibody, 10 mM, Merck Millipore)^58^. Cells were recorded for at least 30 min before and after the 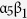 treatment.

### Live cell imaging

Keratocyte migration was visualized via time-lapse microscopy with a Nikon Eclipse Ti-E motorized inverted microscope equipped with ×10 Plan Apo, ×40 Plan Apo (NA 1.45, oil immersion), ×60 Plan Apo (NA 1.45, oil immersion) and ×100 Plan Apo (NA 1.45, oil immersion) objectives and recorded with a Roper QuantEM:512SC EMCCD camera (Photometrics, Tucson, AZ) using NIS Elements Advanced Research 4.0 software (Nikon). Images were recorded every 5 sec. for a total time of 2000 sec. Migrating cells in contact with neighbours were neglected. Selected cells were highlighted and their centroids at each time point recorded. Tracking of the keratocyte trajectories was done with NIS Elements Advanced Research 4.0 software (Nikon, Japan) and analyzed with Origin 8.5 (OriginLab, Northampton, MA).

### Epifluorescence and confocal microscopy

Immunofluorescence stained preparations were observed in epifluorescence with a Nikon Eclipse Ti-E motorized inverted microscope equipped with ×60 and ×100 Plan Apo (NA 1.45, oil immersion) objectives, two lasers (Ar ion 488 nm; HeNe, 543 nm) and a modulable diode (408 nm) and recorded with a Roper QuantEM:512SC EMCCD camera (Photometrics Tucson, AZ) using NIS Elements Advances Research 4.0 software (Nikon). Morphometric analysis (area, perimeter, length, breadth, and shape factor) of focal adhesions was conducted using a custom-made Matlab code and confocal images using small Z-depth increments between focal sections (0.15 μm) were processed using NIS-Elements (Nikon, Advanced Research version 4.0).

### Scanning electron microscopy

Keratocytes were washed in PBS and fixed in a freshly prepared 3% glutaraldehyde solution during 1 hour, as described elsewhere^59^. After fixation, cells were rinsed in PBS and incubated in osmium 1% solution during 1 hour. Cells were then rinsed and incubated in successive baths of increasing concentrations of ethanol diluted in deionized water and finally with a mixture of hexamethyldisilazane/ethanol to allow dehydratation. Subsequently, coverslips were left to dry at room temperature under a chemical hood during 5 to 10 min and mounted on aluminium stubs. Finally, cells were coated with a thin layer of gold in a JEOL JFC-1100E sputtercoater and observed with a JEOL JSM-6100 scanning electron microscope.

### Statistical analysis

Differences in means between groups were evaluated by two-tailed Student’s t-tests performed in Origin 8.5 (OriginLab, Northampton, MA). For multiple comparisons the differences were determined by using an analysis of variance (ANOVA) followed by Tukey post-hoc test. *p < 0.05, **p < 0.01 and ***p < 0.001. Unless otherwise stated, all data are presented as mean ± standard deviation (S.D.).

## Additional Information

**How to cite this article**: Riaz, M. *et al.* Persistence of fan-shaped keratocytes is a matrix-rigidity-dependent mechanism that requires α_5_β_1_ integrin engagement. *Sci. Rep.* **6**, 34141; doi: 10.1038/srep34141 (2016).

## Supplementary Material

Supplementary Movie S1

Supplementary Movie S2

Supplementary Movie S3

Supplementary Movie S4

Supplementary Movie S5

Supplementary Movie S6

Supplementary Movie S7

Supplementary Information
