# Supplementary material for: Persistence of fan-shaped keratocytes is a matrix-rigidity-dependent mechanism that requires α5β1 integrin engagement
Source: Sci Rep. 2016 Sep 28;6:34141. doi: 10.1038/srep34141 (PMC5039689; doi:10.1038/srep34141)
Supplement: Supplementary Information [file srep34141-s8.pdf]

Supplementary Information

Persistence of fan-shaped keratocytes is a matrix-rigidity-dependent mechanism that requires $\alpha_5\beta_1$ integrin engagement

Maryam Riaz[1†], Marie Versaevel[1†], Danahe Mohammed[1], Karine Glinel[2] and Sylvain Gabriele[1*]

[1]Mechanobiology & Soft Matter Group, Laboratoire Interfaces et Fluides Complexes, Centre d'Innovation et de Recherche en Matériaux Polymères (CIRMAP), Research Institute for Biosciences, Université de Mons, 20, Place du Parc, B-7000 Mons, Belgium.

[2]Institute of Condensed Matter & Nanosciences, Bio & Soft Matter division, Croix du Sud 1, box L7.04.02, B-1348 Louvain-la-Neuve, Belgium.

[†]These authors contributed equally to this work

*To whom correspondence should be addressed: sylvain.gabriele@umons.ac.be

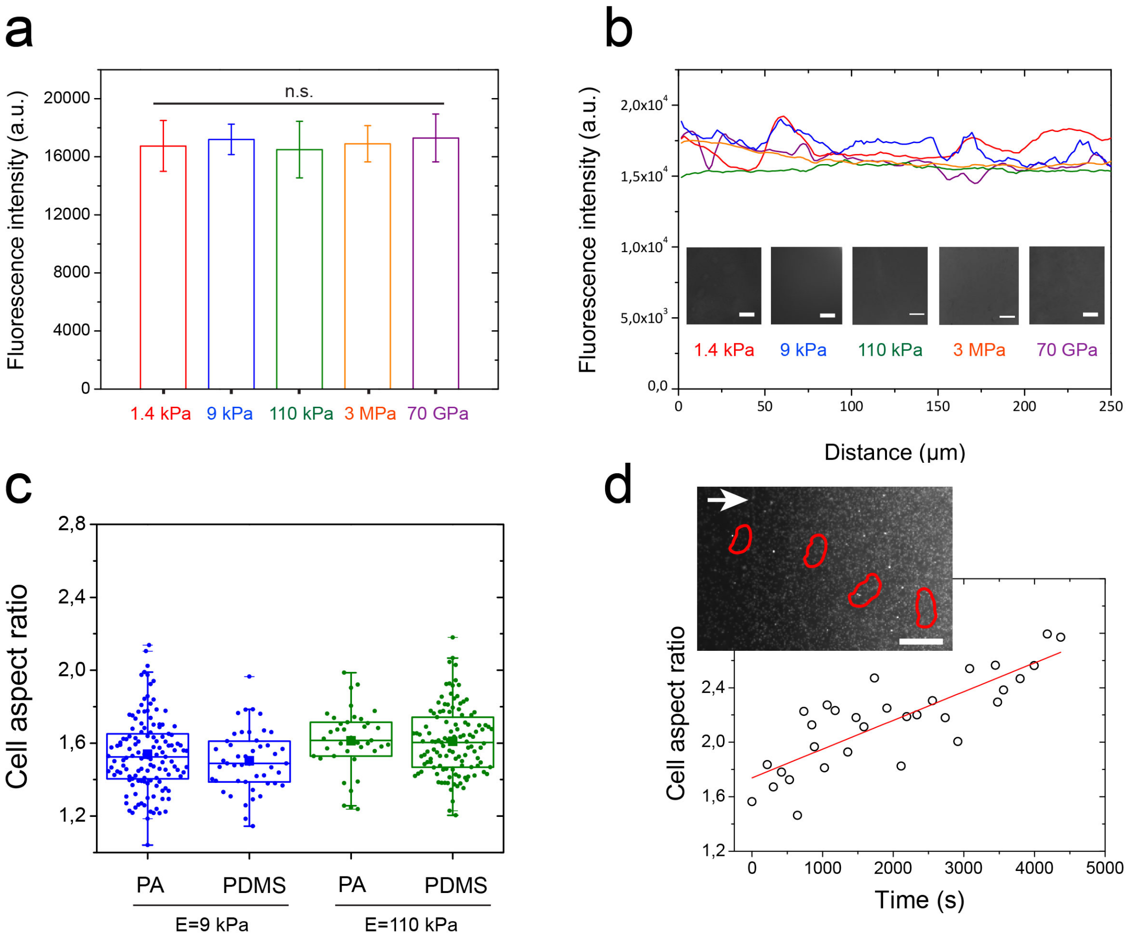

Figure S1. (a) No statistically significant differences in fibronectin density, as measured via fluorescence intensity of TRITC-fibronectin, were observed among PAAm gels of 1.4 kPa (in red) and 9 kPa (in blue), PDMS substrates of 110 kPa (in green) and 3 MPa (in orange) and glass coverslips of 70 GPa (in purple) that were each conjugated with fibronectin at 50 μg/mL. **(b)** Plot profiles of the fluorescence intensity emitted by the TRITC labelled FN after substracting the background fluorescence for PAAm gels of 1.4 kPa (in red) and 9 kPa (in blue), PDMS substrates of 110 kPa (in green) and 3 MPa (in orange) and glass coverslips of 70 GPa (in purple). All scale bars are 50 μm. n.s. non significant. (c) Evolution of the cell aspect ratio of living fish epithelial ketarocytes migrating on 9 kPa (blue datas) and 110 kPa (green datas) matrices. PA and PDMS represented both rigidities for comparison. No significant statistical differences were observed between PA and PDMS of same rigidities. $46 \leq n \leq 113$, Mean ± S.D. $*p < 0.01$ and n.s. not significant. (d) Evolution of the cell aspect ratio

during a typical crossing event of a fish keratocyte from soft to stiff substrate. The inset shows the movement of a single keratocyte at the border between soft (9 kPa) and stiff (230 kPa) regions superimposed on the fluorescent image of beads embedded in the stiffer region of the hydrogel (see Movie S1). Red lines represent the cell boundary. White arrow indicates the direction of gradient from the softer (9 kPa) to the stiffer region (230 kPa). Scale bar is 50 μm.

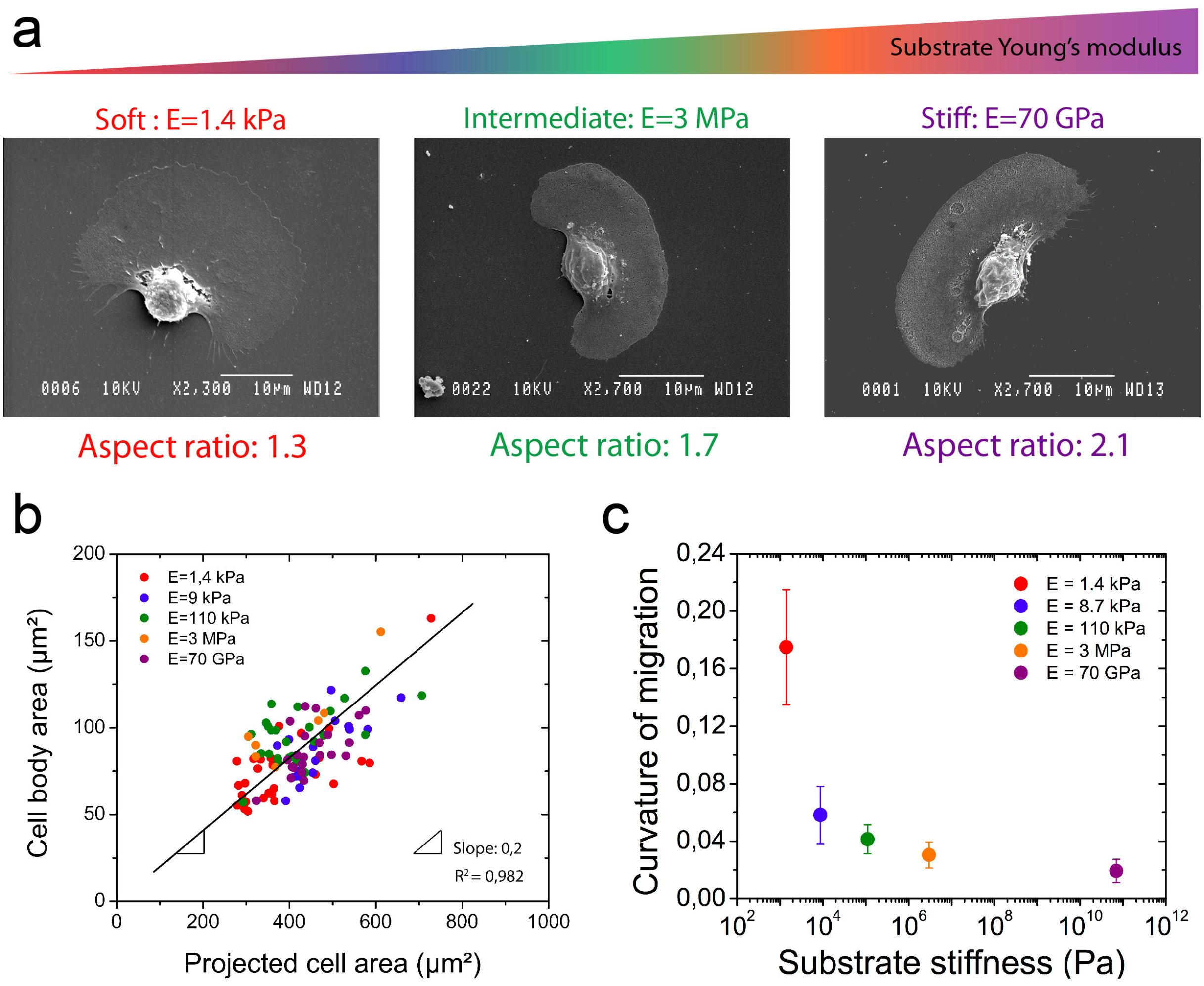

Figure S2. (a) SEM images indicated that fish epithelial keratocytes adopted a rounded morphology on soft (E = 1.4 kPa) matrices, a polarized shape on intermediate (E = 3 MPa) matrix stiffness and a canoe shape on stiff (E = 70 GPa) matrices. Scale bars are 10 μm. (b) Linear evolution of the cell body area as a function of the total cell area for keratocytes plated on FN-coated matrices of 1.4 kPa (red), 9 kPa (blue), 110 kPa (green), 3 MPa (orange) and 70 GPa (purple). The black line corresponds to a linear regression of slope 0.2. (c) Semi-log representation of the evolution of the curvature of migration as a function of the substrate stiffness. $48 \leq n \leq 64$. Mean ± S.D.

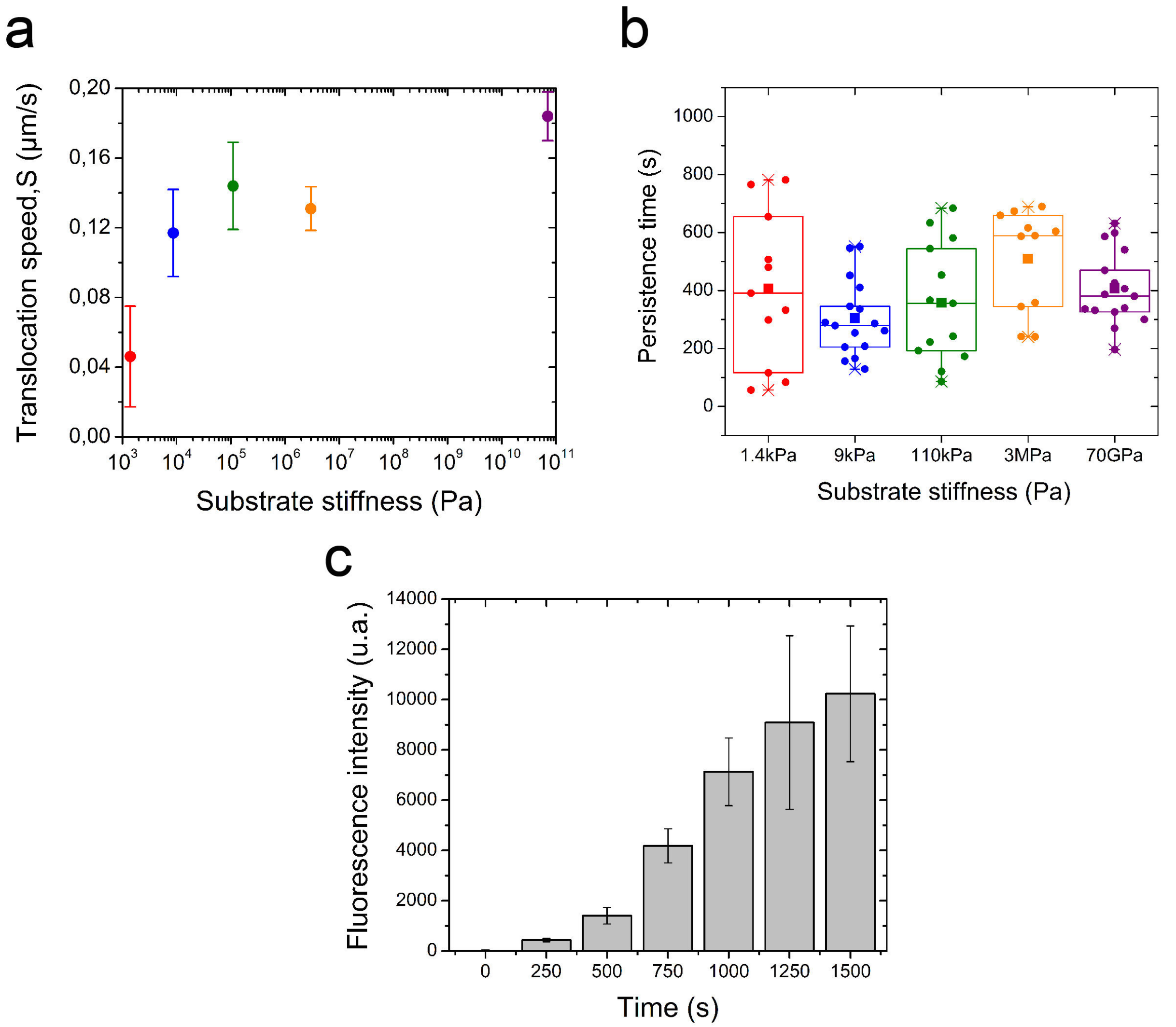

Figure S3. (a) Evolution of the mean translocation speed as a function of the substrate stiffness. (b) Evolution of the mean persistence time, T, as a function of the substrate stiffness. (c) Evolution of the fluorescence intensity of PLL-coated glass substrates after adding TRITC-labelled FN in the culture media at t = 150 sec (n=5).

Movie S1. Time-lapse sequence of an individual keratocyte crossing the border between soft (9 kPa, left) and stiff (230 kPa, right) regions of a polyacrylamide matrix coated with FN. Movie accelerated 90×. Total duration time: 51 min 06 sec. Scale bar is 50 μm.

Movie S2. Cross-sectional reconstruction (*xz*) from a 3D confocal image of the nucleus (DNA in blue) and the actin cytoskeleton (in green) of a single keratocyte plated on a soft FN-coated matrix. Scale bar is 10 μm.

Movie S3. Cross-sectional reconstruction (*xz*) from a 3D confocal image of the nucleus (DNA in blue) and the actin cytoskeleton (in green) of a single keratocyte plated on a FN-coated matrix of intermediate rigidity. Scale bar is 10 μm.

Movie S4. Cross-sectional reconstruction (*xz*) from a 3D confocal image of the nucleus (DNA in blue) and the actin cytoskeleton (in green) of a single keratocyte plated on a stiff FN-coated matrix. Scale bar is 10 μm.

Movie S5. Time-lapse sequence of keratocytes migrating on a stiff PLL-coated surface with an addition of FN in the culture media at t = 150 sec. The frame rate is accelerated 75×. Total duration time: 33 min 15 sec. Scale bar is 20 μm.

Movie S6. Time-lapse sequence of a single keratocyte migrating on a stiff FN-coated surface with an addition of $\alpha_5\beta_1$ antibody in the culture media. The frame rate is accelerated 105×. Total duration time: 01 h 04 min 56 sec. Scale bar is 50 μm.

Movie S7. Time-lapse sequence of a single keratocyte migrating on a stiff VN-coated surface with an addition of FN in the culture media. FN is added at 42 min 50 sec. Total duration time: 02 h 18 min 40 sec. The frame rate is accelerated 250×. Scale bar is 50 μm.
